# Lie Recognition with Multi-Modal Spatial–Temporal State Transition Patterns Based on Hybrid Convolutional Neural Network–Bidirectional Long Short-Term Memory

**DOI:** 10.3390/brainsci13040555

**Published:** 2023-03-25

**Authors:** Sunusi Bala Abdullahi, Zakariyya Abdullahi Bature, Lubna A. Gabralla, Haruna Chiroma

**Affiliations:** 1 Department of Computer Engineering, Faculty of Engineering, King Mongkut’s University of Technology Thonburi, 126 Pracha-Uthit Road, Bang Mod, Thrung Khru, Bangkok 10140, Thailand; sbabdullahi@ieee.org; 2 Zonal Criminal Investigation Department, Nigeria Police, Louis Edet House Force Headquarters, Abuja, Nigeria, Nigeria Police, Abuja 900211, Nigeria; 3 Department of Electrical and Information Engineering, Faculty of Engineering, King Mongkut’s University of Technology Thonburi, 126 Pracha-Uthit Road, Bang Mod, Thrung Khru, Bangkok 10140, Thailand; 4 Department of Computer Science and Information Technology, Applied College, Princess Nourah Bint Abdulrahman University, Riyadh 11671, Saudi Arabia; 5 College of Computer Science and Engineering, University of Hafr Al Batin, Hafar al-Batin 31991, Saudi Arabia

**Keywords:** artificial intelligence, bidirectional long short-term memory, convolutional neural network, computational intelligence, eye aspect ratio, hand gestures, lie recognition

## Abstract

Recognition of lying is a more complex cognitive process than truth-telling because of the presence of involuntary cognitive cues that are useful to lie recognition. Researchers have proposed different approaches in the literature to solve the problem of lie recognition from either handcrafted and/or automatic lie features during court trials and police interrogations. Unfortunately, due to the cognitive complexity and the lack of involuntary cues related to lying features, the performances of these approaches suffer and their generalization ability is limited. To improve performance, this study proposed state transition patterns based on hands, body motions, and eye blinking features from real-life court trial videos. Each video frame is represented according to a computed threshold value among neighboring pixels to extract spatial–temporal state transition patterns (STSTP) of the hand and face poses as involuntary cues using fully connected convolution neural network layers optimized with the weights of ResNet-152 learning. In addition, this study computed an eye aspect ratio model to obtain eye blinking features. These features were fused together as a single multi-modal STSTP feature model. The model was built using the enhanced calculated weight of bidirectional long short-term memory. The proposed approach was evaluated by comparing its performance with current state-of-the-art methods. It was found that the proposed approach improves the performance of detecting lies.

## 1. Introduction 

**Figure 1:**
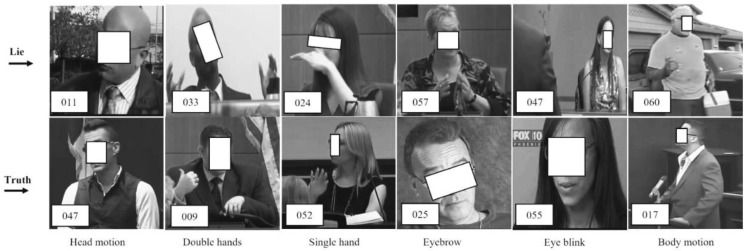
Sample of real-life court trial video data set with involuntary cognitive cues. From the top left corner: (1) lying during the court trial with a forward head motion, (2) lying with double-hand motions, (3) lying with a single hand motion, (4) lying with an eyebrow, (5) lying with an eye blink, and (6) lying with a body motion. From the left bottom corner to the right: (7) truth-telling during the court trial with a forward head motion, (8) truth-telling with double-hand motions, (9) truth-telling with a single hand motion, (10) truth-telling with an eyebrow, (11) truth-telling with an eye blink, and (12) truth-telling with a body motion [1].

On average, every person tells lies at least twice a day [1]. More aggravating is lies presented against others during court trials, police interrogations, interviews, etc., which change the outcome of relevant facts and may lead to wrong judgments or convictions. These problems have inspired the development of computer engineering systems, such as electroencephalography (EEG). Despite the benefits of computer engineering systems for lie recognition, some restrictions exist, such as being cumbersome, which allows a liar to understand that they are being monitored, thus resulting in the presence of deliberate behavioral attitude that can confuse the interviewers. Such deliberate behavioral attitude affects involuntary cues, which mislead the actual results. These involuntary cues comprise facial expression, body language, eye motion, and hand motion, as shown in Figure 1. Each subfigure contains a scene from a court trial video. The scene contains a label in the white box corresponding to the number from the video clip of the original court trial video data set. The scenes from the top left corner to the right show the behavioral attitudes of lying people, while the scenes from the bottom left corner to the right show the behavioral attitudes of truth-telling people. Addressing the problem of learning human involuntary cues, recent research in the field of image processing/CV and machine learning reshapes computer engineering systems into machine learning-based (ML) systems [2,3,4]. ML-based systems can learn tiny facial marks [4] and behaviors in connection with body motion, as well as hand gestures [5], therefore making lie recognition suitable via CV and ML techniques. However, a combination of two or more human involuntary actions (known as cognitive cues) provides good results at some expenses [6]. Therefore, deep learning with CV features, such as bidirectional long short-term memory (BLSTM), has advanced with appreciable performance, although only a few examples have appeared in the literature [1]. However, the weights of BLSTM do not highlight the key information in the context, which leads to information redundancy when learning long video sequences [7], as well as insufficient recognition accuracy and model instability [1].

The recognition accuracy of these methods [1,4] is low because some multi-modal involuntary cues and their complementary information are missing; thus, their accuracy needs to be improved since involuntary cues, such as those shown in Figure 1, are significant factors in determining people’s behaviors while giving testimony during court trials or investigations. These involuntary cues are difficult to capture by using classical technique. Thus, deep learning methods are the suitable choice. However, deep learning methods provide a huge amount of information that is sometimes irrelevant to lie recognition. Uncertainty about the type of multi-modal information to be used for lying recognition remains a key factor. Thus, we improve this process by highlighting the key information of multi-modal features by proposing multi-modal spatial–temporal state transition patterns (STSTP). It is found that the highlighted multi-modal STSTP information provides a sound basis for lie recognition under real-life court trial videos and paves the way for the development of explainable and principled tools. Inspired by these results, we propose spatial–temporal state transition patterns based on involuntary actions of lying and truth-telling persons.

The organization of the paper proceeds as follows: the literature review is described in Section 2. Section 3 deals with the process of the proposed methods and the adopted algorithms. Section 4 describes the data source and its characteristics. It also describes the data preprocessing and feature engineering. Section 5 presents the results of the proposed models and performance evaluations. It also discusses and interprets the main results of the conventional methods. Finally, Section 6 concludes the paper. The main contributions of this article can be summarized as follows:

(1) This study designs a state transition pattern vector based on STSTP to model the involuntary cognitive cues of the hand, body, and eye-blinking motion of a lying or truth-telling person.
(2) This study presents lie recognition with multi-modal STSTP based on hybrid ResNet-152 and BLSTM.
(3) The proposed approach controls redundant features and improves computational efficiency.
(4) The performance evaluation indicates the superiority of the proposed approach when compared to classical algorithms.
(5) This study computes facial involuntary actions using the EAR formulation, while complete body motion is computed using optical information to distinguish between involuntary lying and truthful cognitive indices.
(6) This work demonstrates empirical evidence of an improved police investigation/court trial process with an automatic system, compared to a single and manual lie recognition system.

## 2. Literature Review

### 2.1. Eye Blinking Approach 

Eye blinking is an involuntary cue during lying or truth-telling actions; however, it is a valuable index to enhance effective recognition. The eye-blinking cues of a lie are hard to learn during a cross-examination or a court trial. Although complex techniques are in use to record eye blinking, such as eye trackers, these techniques need a biomarker and complex data interpretation. Therefore, RGB videos from computer vision (CV) provide a flexible data set for the recognition of lies. CV allows an algorithm to be built without the need for a biomarker and/or complex data interpretation support. Eye-gaze lie systems, such as that of Bhaskaran et al. [8], propose eye-gaze features based on dynamic Bayesian learning. This method was reported to achieve an accuracy of 82.5% in learning distinct features between deceit and non-deceit cues. The major limitation of this work includes failure to reflect real-life scenarios, such as a suspect or witness wearing glasses or showing flicking an eyebrow motion. Proudfoot et al. [9] proposed eye pupil diameter using a latent growth curve modeling technique to capture changes in the eyes of the suspect and complainant, while George et al. [10] evaluated the number of eyeblink counts and their duration among lying and truth-telling persons. The former study finds that significant changes occur when a person is telling lies, while the latter study can conclude when a lying person is pressurized. The advantage of the work by Avola et al. [4] is that it highlights the benefits of extracting macro- and micro-expressions (MME) during police interrogation, cross-examination, and court trials. Macro- and micro-expressions of the face are built in an ensemble fashion. Therefore, it can be observed that a truth-telling or lie-telling person employs various body cues (multi-modal cues) to express themselves, as shown in Figure 1; thus, single-body cues are not sufficient to discriminate lies from facts.

### 2.2. Multi-Modal Cue Approaches 

An automated multi-modal lie recognition system can allow the building of a system with potential behavioral cues to distinguish a lie from the truth [11]. The work by PrezRosas et al. [12] exploited verbal and non-verbal indices to detect court verdicts with decision trees and random forests. Abouelenien et al. [13] demonstrated the performance of cross-referencing physiological information with a decision tree and majority voting strategy, while Karimi et al. [14] exploited visual and acoustic cues using large margin nearest neighbor learning. Wu et al. [15] considered visual, audio, and text information in unison to compare and select the best classifier among decision trees, random forests, and linear SVM. Rill-Garcia et al. [16] jointly combined visual, acoustical, and textual indices using SVM to evaluate the effectiveness of the combined information. Krishnamurthy et al. [17] utilized a 3D CNN for feature extraction, and classification was conducted using multi-layer perceptron. This work demonstrates the effectiveness of multi-modal deep learning cues. 

Furthermore, hand features are very stable cues for identifying human actions and intentions, as reported in the literature [3,18,19]. Lu et al. [20] extracted hand and facial features using color 3-D LUT, which are further utilized with blob analysis to track head and hand motions (behavioral state). Their method needs to be improved to avoid complex segmentation and long processing time. Meservy et al. [11] extracted hand and facial features using color analysis, eigenspace-based shape segmentation, and Kalman filters. The major limitation of this method is user invariability. Avola et al. [1] extracted hand features from RGB videos using OpenPose. In their method, the hand is represented using 21 finger joint coordinates per frame along with acceleration and velocity. In addition, their method calculates hand elasticity and openness to observe hand behavior while lying or speaking the truth. Mut Sen et al. [5] proposed visual, acoustic, and linguistic modalities. This method designs automatic and manually annotated features using a random seed, and the features are validated using different classifiers in semi-automatic and automatic modes. The best results are obtained from the semi-automatic system with artificial neural network classifiers. The work in [5] proposes a multi-feature approach based on subject-level analysis. The features are detected manually, which affects the performance. Most of the current best works achieve the best result via deep learning methods. However, eyebrow, eye blinking, and optical flow of involuntary information are not utilized by those methods; thus, the current challenges have not been properly addressed.

## 3. The Proposed Conceptual Framework

**Figure 2:**
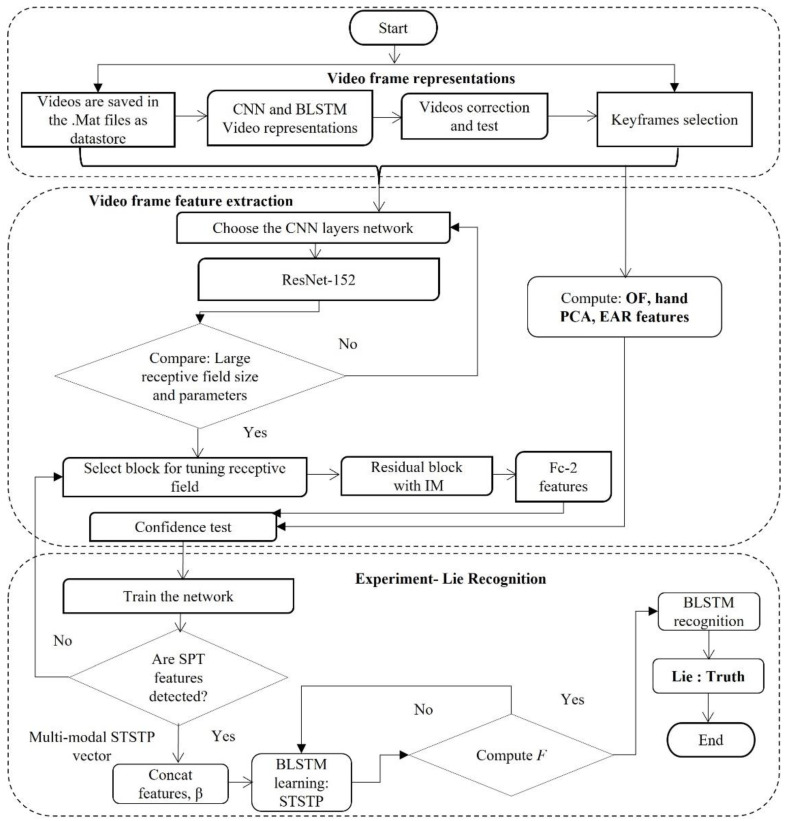
Proposed method.

Each process for the multi-modal STSTP real-life court trial video models for lie recognition is detailed in the following sections. This study investigated and proposed three spatial–temporal involuntary cognitive cues as a state transition pattern vector: the hand, body motion, and eye blinking (eye aspect ratio (EAR) and eyelashes) staging video information of fifty-six suspects and witnesses during real-life court trials, who were either lying or truth-telling. In the first stage, the court trial videos were selected by a keyframe selection threshold method, and the feature indexes were sorted according to the frame significance. This study sorted the frames of each person by using the EAR, and the first ten frames was randomly sampled. From these frames, a total of 20 hand joint poses were located using principal component analysis (PCA), and body motion was detected using optical flow information (OF). This information was combined as three spatial–temporal state transition patterns (STSTP) that are significantly associated with a person either lying or telling the truth, which were verified by the curve fitting tool. In the second stage, the fully connected layer at the 20th layer was initialized using the effective weight of ResNet-152 to achieve the best spatial feature (SP) extraction. In the third stage, the keyframe selection approach was introduced to optimize the parameters and weights in the BLSTM network training process to improve the stability and performance of the proposed STSTP model. Finally, a lie recognition model of a person who is either lying or telling the truth with the STSTP based on ResNet-152-BLSTM is established. The whole process is depicted in Figure 2.

### 3.1. Video Frame Representation from the Proposed Method 

#### 3.1.1. Convolutional Neural Network-Based Video Representation 

Convolutional neural network (CNN) has excelled in many image and video recognition problems due to its local dependencies and scale invariance. However, in CNN, the window size is also investigated, where it has been found that a larger window size does not lead to a better performance in all cases of cross-examination classification. We designed a wide convolution to control CNN feature maps. In this case, we exploited the benefits of the residual network (ResNet) layer as a CNN variant. We adopted the ResNet output from the Conv1-2 layer as our feature extractor. The output of the Conv1-2 layer is utilized in the average pooling layer for the recognition and classification by the BLSTM layers. Let the video set of lie and truth be $L=c_{1,\;}c_{2,\ldots ,}c_{n}$, with $c_{i}$ *∈* $R_{s}$, where s denotes the cognitive index size. The total number of cognitive indices considered in each window for the convolution operation is denoted as o; then, the width of the convolution kernel is maintained as in the cognitive embedding size. Therefore, the convolution kernel can be obtained as $e\in R^{\left(o\times s\right)}$. However, each window slide gives a convolution output as follows:

(1) $$y_{i}=ReLU\left\{e\cdot c_{i:i+o-1}\right\}+q,$$

where $ReLU,c_{i:i+o-1},\mathrm{and}\;q$ denote the nonlinear activation function, the number of cognitive indices taken in each convolution operation, and the bias term q∈R, respectively. The padding parameter is set to be the same as the length of the video set L, which is set to be n. The stride size is D. Therefore, the convolution output becomes $y=\left[y_{1,}y_{2,\ldots ,}c_{n}/D\right]$. Then, the pooling layers perform maximum pooling operation, as shown in Equation (2). The pooling operation includes sliding a 2D filter per channel of feature map and summarizing the features spreading within the region covered by the filter. Thus, the dimensions of the output obtained after a pooling layer are given as follows:

(2) $$P_{max}=\frac{H_{f}-U+1}{D\times \left|\left(W_{f}-U+1\right)\right|D\times C_{f}}$$

where $H_{f},\;W_{f},\;C_{f},\;U,$ and D denote the feature map height, width, number of channels, filter size, and stride length, respectively. In addition, padding layers K are added such that the output image has the same dimensions as the input image, as given below:

(3) $$\left\{\left(I+2K\right)\times \left(I+2K\right)frame\right\}*\left\{\left(U\times U\right)\ker\;n\;el\right\}↪\left\{\left(I\times I\right)frame\right\}$$

where $K=\frac{\left(U-1\right)}{2}$.

Finally, the total output of the fully connected layer for the cognitive CNN is obtained as follows:

(4) $$CNN=\to \left\{\left[G\right],\Lambda =-1\right\}$$

where →,G, and Λ denote the concatenation operation, the output of the layer, and the procedure of dimension/size splicing, respectively.

#### 3.1.2. Bidirectional Long Short-Term Memory-Based Video Representation 

Long short-term memory (LSTM) architecture was chosen because of its capability to learn long-term dependencies in video sequences [21]. LSTM with backward and forward memory cells is known as BLSTM, which can effectively preserve the semantic information of longer sequences. A single BLSTM network for multi-modal feature recognition leads to low accuracy and over-fitting, especially when learning complex video sequences. To address this problem, stacking more than one BLSTM unit, such as in [7,21], enhances the recognition performance of multi-modal features. Therefore, inspired by these works, this study designed a BiLSTM architecture using two BLSTM units. This architecture allows the achievement of high-level sequential modeling based on the selected STSTP features. Finally, the splicing of the output vectors of the forward and backward BLSTM units is performed, and the feature vectors with bidirectional semantics are the output of the BiLSTM layer, as given in Equation (5). The BiLSTM fails to show the importance of key information in context during computation, and it causes information redundancy when dealing with long STSTP sequences. Therefore, we set up a threshold value to average the entire STSTP features that can effectively highlight important cognitive features to ease the BLSTM learning. The key feature extraction according to the threshold method is highlighted in Equations (6) and (7). Finally, a categorical label is designed from the two classes LIE and TRUTH, which are further classified using the Softmax layer at the last LSTM layer with FC to better analyze lying and truth-telling persons. Therefore, the final BLSTM output is obtained as follows:

(5) $$BLSTM=\left\{\vec{h_{t}};\;h_{t}\right\}\in R^{F}$$

where $h_{t}$ and F denote the backward and forward hidden layer output information and the key features, which can be obtained as follows:

(6) $$F=\left\{\begin{array}{c} 0,\;if\;K_{i}<T\\ F,\;if\;K_{i}\geq T. \end{array}\right.$$

#### 3.1.3. Scheme to Control the Network Saturation 

The strategy used in this paper to control the hard learning of BLSTM weight is to create an empty set matrix X from the pool of generated STSTP cognitive cues Y by the LSTM neurons. In such a case, X is fed from the pool and increments the search criterion J. The most significant features/context Y from the STSTP is fed into the corresponding set in each cell in the matrix, as described in step 1 of Algorithm 1. However, the worst STSTP value from the set is dropped if *J* increments, as described. Then, the newly created matrix set vector is the sub-partition set of the classes L (lie) and T (truth). The newly created matrix is fed into the BLSTM layer for recognition in step 10. Aiming to control the BLSTM learning instability, which can lead to poor recognition results, we further tuned the learning rate and batch size of the BLSTM network; the formulated learning loss function is explained in Section 4.3.
**Algorithm 1**: Guided-learning algorithm1:    **start** 2:    **set** $V\left[row,col\right]=X$ in Equation (1) {create matrix} 3:    **set** $Y=\left[y;{|}_{j=1}:N\right]$ in Equation (1) {STSTP sequence} 4:    **set** $X_{k}=\left[x_{l{|}_{l=1}}:D,x_{l}\epsilon Y\right]${output} 5:    **set** $X_{0}=0$ {Initialization} 6:    **Evaluate** Equation (6) {selection} 7:    **for** each k=k+1 do 8: **repeat** 9:        **if** $J(X\_k|\;\max\_\{x\epsilon Y|x\_k\}J(X\_k|x)>J\left(X\_k\right))\;$**then** 10:    **Evaluate** Equation (5) 11:    **else** 12:    **go to**
*STEP 1* 13: **until** Equation (14) converge 14. **return** $\left\{K=K+1\right\}$ 15: **end**

### 3.2. Video Frame Feature Extraction 

#### 3.2.1. Frame Extraction Strategy 

First, we adopted the strategy of obtaining keyframes *F* to control the processing time. Equation (6) searches for any similar frames within the sequence for each performance. The equation further finds the difference between the sum values of each RGB image and its adjacent RGB images to distinguish between similar frames using a threshold value. In Equation (7), V denotes the video frames, P denotes the parameter to set a threshold for K video frame representation, and T denotes the threshold used as given in Equation (6).

(7) $$T=\frac{P}{V}\sum_{i=1}^{v}\left|K_{i}-K_{i+1}\right|$$

#### 3.2.2. Optical Features of Real-Life Court Videos 

Motion provides multi-contextual information, which highlights the need to understand visual actions, etc. Estimating the motion of every pixel in a sequence of court trial RGB video frames is an effective way of using the OF. The OF is a method for RGB video motion estimation. The OF represents a sparse vector, where a displacement vector is devoted to a specified pixel position that points to where a pixel can be found in another image [22]. Since the Lucas–Kanade schema can track points on a moving video frame precisely, we employed a function cv2.goodFeaturesToTrack() to compute the points within the court trial videos. A value of 100, 0.3, 7, and 7 was chosen for the maximum corner points, quality level, minimum distance, and block size, respectively. For each video, we tracked the initial frame and captured its corner points using the Lukas schema cv2.calOpticalFlowPyrLK(). The following parameter values were chosen for the Lukas schema: 15 and 4 for the window size and the maximum level, respectively.

#### 3.2.3. Hand Features 

**Figure 3:**
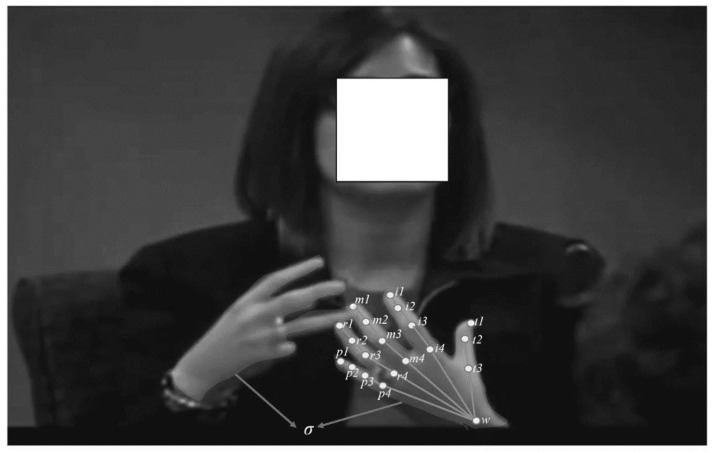
Hand features using PCA.

We extracted 21 hands joint information from 5 conventional fingers, palm, and wrist joints of a single hand, while information from 42 hand joints was extracted in the case of double hands. Each finger joint was computed according to its anatomical definition, as shown in Figure 3. We further ignored the palm center pose as it was less important to our gestures. Finally, we have 20 and 40 hand joint poses for single and double hands, respectively. The thumb is labeled as *t1* to *t3*. The index finger is labeled as *i1* to *i3*. The middle finger is labeled as *m1* to *m4*. The ring finger is labeled as *r1* to *r4*. The little finger is labeled as *p1* to *p4,* while the wrist is labeled as *w*. In the case of double hands, we consider the relationship between the two hands, which is denoted as σ in Figure 3. However, some hand motion information is also obtained from the OF information. We further obtained hand information within the video frames using principal component analysis (PCA). In PCA, each frame coordinate is transformed into eigenvector space, with the mean of the frame being mean(f), which is modeled into a transformed frame, g, using the transformation matrix B to obtain Karhunen–Loeve transform, as defined in Equation (8). It becomes clear that the vector rotation makes a huge amount of eigenvector spread within the features. Justification for the hand shape and orientation features within each frame stems from their ability to show orthogonality.

(8) $$g=B\left(F-mean\left(f\right)\right)$$

#### 3.2.4. Eye Aspect Ratio Features 

**Figure 4:**
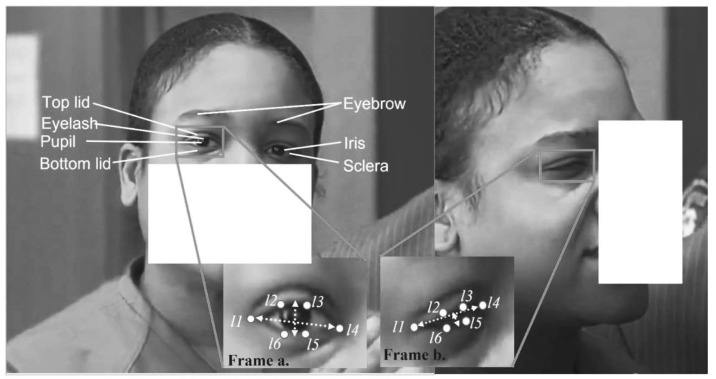
Features of EAR. Frame (**a**) Shows the computed EAR features while eye is open, frame (**b**) show the computed EAR features while eye is closed [1].

In this method, eye poses are computed as eye-blinking features using the algorithm designed by [23]. The eye-blinking poses are known as eye aspect ratio (EAR) features. We were able to capture eye-blinking poses, such as eyebrow, bottom eyelid, top eyelid, etc. We localized the initial face position, and then we further bounded the point of interest (POI). We defined the circular Hough transform (CHT) to locate and write the iris in the POI. During an eye blink, the CHT displays approximate null information, while the centroid rotation coordinates are considered, as shown in Frame (b) of Figure 4. The POIs are tracked among the consecutive frames with the keyframe method. This method allows us to obtain frames that have the point with the maximum likelihood value. These frames have the POI denoted from point *L*. The point for a given time t consists of six cues $L_{t}=\left(l1_{t},l2_{t},l3_{t},l4_{t},l5_{t},l6_{t}\right),$ denoting the *x* and *y* coordinates of eye rotation, as shown in Frame (a) of Figure 4. *Lt* is mapped by a Gaussian distribution enclosed in *Lt* − 1. The most likely POI is learned and corrected with adaptive histogram equalization. The bounding box is used to obtain the eyebrow region when the subject blinks their eyes. With this information, we finally computed eye aspect ratio (EAR) using the following equation:

(9) $$EAR=\frac{|\left|l2-l6\right||+\left|\left|l3-l5\right|\right|}{2\left|\left|l1-l4\right|\right|}$$

where l1,…,l6 denotes the 2D poses between top and bottom lid blinking. 

We computed the accuracy of the EAR detection using the error metric obtained by Equation (15). This metric shows how effective the point of interest (POI) of each face has been detected, as labeled in Figure 4. In Figure 4, the eyebrow features are saved in the vectors $\left(V1,\ldots ,V3\right)$ format so that the significance of each data point is computed with the curve fitting *nnftool* and measured using the $R^{2}$ metrics to achieve the best features, as illustrated in Table 1. In addition, the EAR features are saved in vectors $\left(Var1,\ldots ,Var3\right)$, where an EAR vector is used as the dependent variable while the three vectors are used as the independent variables; thus, the relationship between the vectors is computed with *nnftool* and the significance of the results is measured using $R^{2}$, as illustrated in Table 2. We performed a statistical confidence test to observe the contributions of the relationship between the eyebrow and eye-blinking features in recognizing involuntary cognitive cues. The test was performed using a curve fitting tool and measured using the $R^{2}$ metrics obtained from Equation (16). The results of the test are presented in Table 3. The test shows high values of $R^{2}$, which demonstrates a good relationship among the features.

### 3.3. Multi-Modal Spatial–Temporal State Transition Pattern Feature Vector 

**Figure 5:**
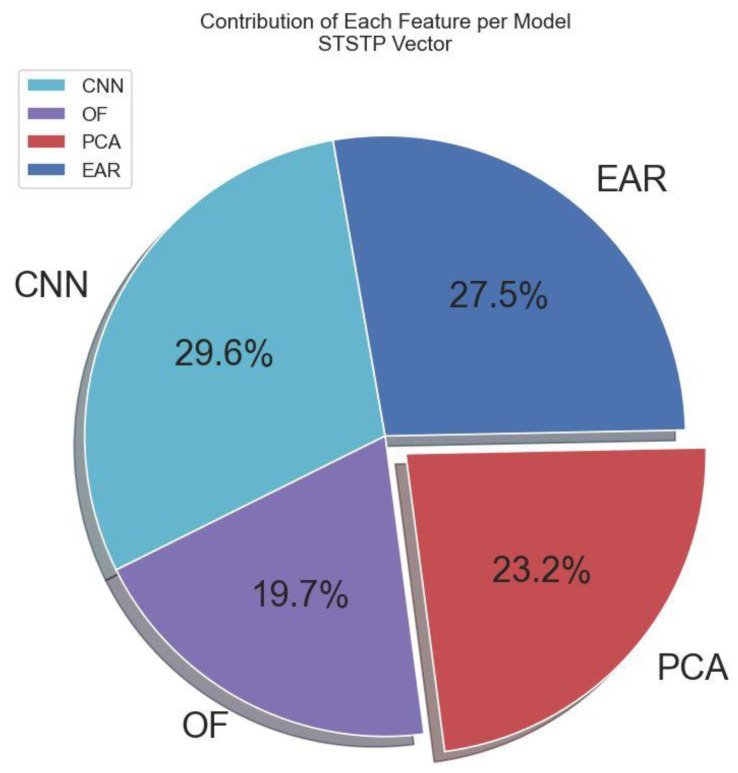
Percentage contribution of each feature in the proposed STSTP model.

In this section, we simply augment each computed vector and matrix in Section 3.2.1, Section 3.2.2, Section 3.2.3 and Section 3.2.4. This is because either CNN, OF, PCA, or EAR feature is not sufficient to decide the action of a suspect, witness, and/or complainant during interrogations, court trials, and cross-examinations. We further use the simple vector operation to combine the spatial information (pixel-level features) from the CNN layers with the augmented (OF, PCA, EAR) features as a single multi-modal STSTP vector. The contribution of each feature per STSTP vector for the utilized method is depicted in Figure 5. The multi-modal STSTP vector is fed into the two enhanced BLSTM layers to generate temporal dependencies with respect to time series. The multi-modal STSTP vector β is finally obtained from the simple vector concatenation as follows:

(10) $$\beta =concat\left[CNN,OF,PCA,EAR\right]$$

### 3.4. Qualitative Analysis of Real-Life Court Trial Videos 

**Figure 6:**
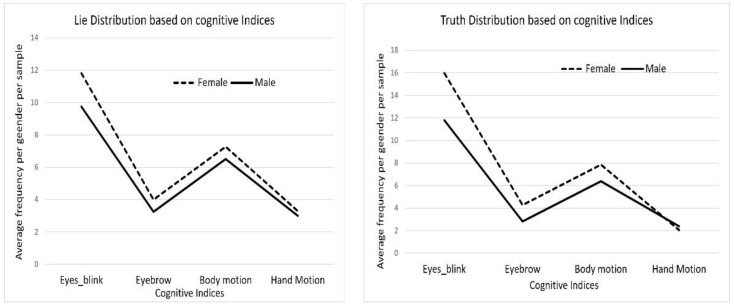
Statistics of the person population based on the cognitive indices.

This study employed both deductive and inductive approaches in qualitative court trial video analysis. We constructed a deductive framework with the main categories as the presentation of the video results, including the hand gestures, eye blinking, and body motion, and the presentation of the filtering options. We independently performed an inductive analysis with the sub-categories as the number of females and males per scene in the videos, the number of times each person performs an involuntary cognitive index, and the body part(s) employed to perform each index, as well as the occurrences via descriptive statistical method and clustering. It is generally observed in Figure 6 that all involuntary cognitive indexes are higher in females, as indicated in the black dotted lines, than in males, as denoted with the black solid lines, except for hand motion which is lower when females are being truthful. Additionally, the blinking rate is generally higher than all other cognitive indexes regardless of gender, followed by body motion and eyebrow, while hand motion is the least utilized by both genders. However, the cognitive indexes in males do not portray appreciable changes, except in the blinking rate which increases when they are making truthful testimony. 

## 4. Experiment 

The proposed multi-modal STSTP with the ResNet-152-BLSTM method is experimentally validated using a real-life court trial video data set. The algorithms are built on the Python 3 and MATLAB R2022a platforms. The platforms are run on a PC equipped with Window 8, CPU intel Core i7, and RAM 8. In this section, the experimental validation is achieved from the following processes: data study, confidence test on the real-life court trial data, recognition of STSTP, selection of network parameters, and finally, performance evaluation metrics. These processes are further elaborated below. 

### 4.1. Data Study 

**Figure 7:**
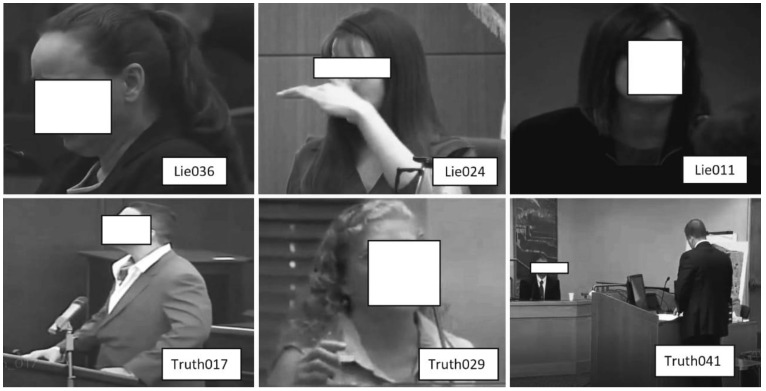
Some samples of noisy frames of court trial videos [1].

The data set is adopted from [1]. This data set consists of one hundred and twenty-one court trial videos. Unlike [1] where they had selected only videos that applied single hand or double hands because of human peculiarities, in this work, we employed the complete 121 videos. The data set was created using a template based on three court trial outcomes: guilty, non-guilty, and exoneration. These three outcomes were labeled as truthful or deceptive. We clustered the truthful cues from exoneration and disposition, which were agreed by the police to be true, whereas the lie or deception cues were clustered from the declarations in favor of the guilty suspects. We confirmed the average duration of the videos to be twenty-eight seconds (28 s), which were filmed by twenty-one females and thirty-five males, respectively. However, in some videos where hand(s) appears, we applied the suitable hand detection algorithm to extract local and global features; where no hand information appears in the clip, the algorithm is written in such a way that the csv files are zero-padded, as shown in Figure 7. In situations where the eyes of the subject are readily available, we applied the eye detection algorithms and the EAR computation mechanism to extract eye and some facial cognitive indices; where such gestures are absent, the csv files are zero-padded. In addition, we further utilized the optical information of each frame to obtain motion features. We split the data into sixty videos to contain truthful scenes, while sixty-one videos contain lie or deceptive scenes. For effective evaluation of the proposed features, we employed k-fold validation with *k* = 10.

### 4.2. Confidence Test 

We obtained a huge number of features from the fully connected layer but features with high confidence scores can improve the computational speed of ResNet-152-BLSTM learning and avoid ignoring important variances [3]. The confidence score of all features is illustrated in Table 4. Since the concatenated STSTP vector generated by the weight of the BLSTM (hidden layers) $\beta_{i}$ contains cognitive cues $h_{i}$, the feature selection *M* can be treated as a maximization problem, using the following formula:

(11) $$M=\underset{{\{}_{\beta \epsilon {\left[0,1\right]}^{{\{}^{i}}}}{\max}\left\{\frac{\sum_{l=1,u=1}^{i}{\left(h_{i}\beta_{i}\right)}^{2}}{\sum_{l=1}^{i}\left(\beta_{i}\right)+\sum_{u\neq 1}^{i}2\times \left(h_{l}\beta_{l,u}\right)}\right\}$$

Features with a big confidence score are considered less significant; thus, we selected STSTP features with high significance among consecutive features. Thus, feature weight was obtained using Algorithm 1 and fed into the Softmax layer for classification.

### 4.3. Recognition of Spatial–Temporal State Transition PatternTransition

We fine-tuned the ResNet-152 network using the output of the last CNN layer (fc-2) which is initialized from the standard CNN layers as described in Table 5, to extract the pixel-level features from the RGB video frames. The ResNet has a depth of 152 layers [14]. From these layers, the residual stage is obtained via identity mapping (IM). However, IM is realized in the residual block via skip connection, thus having the ability to bypass nonlinear transformation. This strategy allows an increase in the network depth without optimization or hard learning. We generated a datastore that reads all the videos and automatically resizes them into 224 × 224 for batch learning of huge video sequences. The 224 × 224 frame size enables this method to preserve memory usage, computing time, and spatial resolution. Since our videos are in RGB format, we chose the channels as 3. This network chose 32 court trial video frames so that the datastore could output each time to exploit more temporal information. The *createFileDatastore* function was used to configure the datastore and to load the court trial videos to Resnet-152. The ResNet-152 models were trained on 121 court trial videos. The ResNet layer extracts geometrical hand and body features. We adopted the output from the 20th layer as our feature extractor as explained in Table 5. The output of the 20th layer was used as the Conv1-2 and replaced with global average pooling which is fed into the BLSTM layer for sequence time-dependent generation as described in Table 6, which was further utilized for the recognition and classification by the Softmax layers. The overall output of the ResNet-152-BLSTM network was obtained by a simple vector concatenating both inputs of the two networks, as given in Equation (12).

(12) $$ResNet-BLSTM=\to \left\{\left[ResNet,BLSTM\right]\right\}$$

The information obtained in Equation (12) was utilized as the input to the categorical probability $\bar{M}$ for the classification of cognitive indices with encoded labels *M* as follows:

(13) $$\bar{M}=soft\max\left\{\Phi_{g}\cdot ResNet-BLSTM+q_{g}\right\}$$

where $\Phi_{g}$ and $q_{g}$ denote the weight and the bias matrix of the average pooling layer, respectively as shows in Table 6. We chose Softmax cross-entropy for the loss function χ throughout the BLSTM network training as follows:

(14) $$\chi \left(\bar{M},M\right)=-\sum_{i=1}^{f}M_{i}\cdot \log\bar{M}$$

### 4.4. Selection of Network Parameters 

See Table 7 for the desired values of each deep learning network design. 

### 4.5. Performance Evaluation Metrics 

We trained the proposed STSTP with the ResNet-152-BLSTM method using the k-fold splits of the data set for both training and testing. The proposed STSTP with ResNet-152-BLSTM outperformed the classical BLSTM, FV-BLSTM, and CNN-BLSTM. The letters used for the metrics are defined as *Oi*, *O’i*, *S*, *θ*, *σ 2 r*, *Oa*, *U*, and c, which denote the original template of the image POI, the estimated POI information, the number of frames, the params., the mean, the POI being considered, and the Euclidean distance between the eye centers, respectively. 

#### 4.5.1. Eye Aspect Ratio Detection Error

This study computed the accuracy of the EAR detection using the error EARerr metrics, as explained in Section 3.2.4. This metric shows how effective the point of interest (POI) of each face feature has been detected, as labeled in Figure 4. The EARerr describes the average relative landmark localization error. The EAR detection error is obtained by using Equation (15), where *Oi* denotes the original template location of landmark I within the video image, *Oi*’ denotes the predicted landmark location by the EAR estimator, *S* denotes the number of landmarks, and *U* denotes the Euclidean distance between the eye centers within the video image. We computed a cumulative histogram of the EARerr for a point of twelve landmarks of the eyes. We also computed the interface landmarks (that is, eyebrow), but we considered the intra-face for eye landmarks only.

(15) $$EAR_{err}=\frac{100}{cU}\sum_{i=1}^{S}\left(O_{i}-O_{i}^{\prime }\right)$$

#### 4.5.2. Coefficient of Determination

This study computed the *R*^2^ using the sum of the square errors of the fitting model $\sum_{i=1}^{S}{\left(O_{i}-O_{i}^{\prime }\right)}^{2}$ and the total sum of the squares $\sum_{i=1}^{S}{\left(O_{i}-O_{ai}^{\prime }\right)}^{2}$. The sum of the square errors is computed from the sum of the squares of the original template of the image POI values and the estimated POI values. The total sum of the squares is computed from the sum of the squares of the POI being considered and the mean value. If the curve fit is good, the sum of the square errors is smaller than the total sum of the squares. The general equation for computing the *R*^2^ is as follows:

(16) $$R^{2}=1-\frac{\sum_{i=1}^{S}{\left(O_{i}-O_{i}^{\prime }\right)}^{2}}{\sum_{i=1}^{S}{\left(O_{i}-O_{ai}^{\prime }\right)}^{2}}$$

#### 4.5.3. Accuracy 

We computed the average recognition accuracy (Acc.) for the performance of the proposed multi-STSTP ResNet-152-BLSTM from the confusion matrix [21]. The confusion matrix contains the correct classified lie and truth within, below, above, and at the diagonal of the entries. The classes are estimated from the possible, available developed STSTP models. Each class is computed based on the frequency of occurrence of the features in the STSTP model. Then, the estimated features are plotted in a matrix according to the four major entries. We defined $a_{1},a_{2},b_{1},\mathrm{and}\;b_{2},$ which denote true positive, true negative, false positive, and false negative, respectively, as the four major confusion matrix entries. The average recognition accuracy is obtained as follows:

(17) $$Acc.=\frac{a_{1}+a_{2}}{a_{1}+a_{2}+b_{1}+b_{2}}$$

The true positive rate or the sensitivity of the model to precisely recognize the involuntary cue of lie during the recognition of all court trial features is computed using the following equation:

(18) $$true\;positive\;rate=\frac{a_{1}}{a_{1}+b_{2}}$$

The false positive rate or specificity metric is computed to show that the STSTP model recognizes the non-lie cue during the recognition of all negative court trial features as follows:

(19) $$true\;negative\;rate=\frac{a_{2}}{a_{2}+b_{1}}$$

## 5. Results and Analysis 

In this section, we present the performance results of the STSTP with ResNet-152- BLSTM. This study evaluated the performance of the ResNet-152-BLSTM based on the STSTP vector from real-life court trial videos in seven different cases. The seven cases were explored using the proposed STSTP models. As shown in Table 8, the first model was developed using the STSTP vector of optical flow information (STSTP-OF), known as model 1. The OF feature vector comprises the body motion information at both local and global levels, as explained in Section 3.2.2. Model 1 achieves an average recognition accuracy of 71.39%. The second model was developed using the computed features of the hand using the PCA method, as described in Section 3.2.3. However, these features were further built to exploit their time-series dependency, and the model is known as Model 2, as shown in Table 8. Model 2 returns an average recognition performance of 59.87%. This accuracy rate demonstrates that the hand features alone are insufficient to recognize the two classes of Lie and Truth. The third model was developed using the computed EAR features, as described in Section 3.2.4, and this model is known as Model 3. Model 3 achieves a recognition accuracy of 61.25%. It is shown that the EAR features are more stable as a cognitive index in lie recognition than the hand features. We developed the fourth model (Model 4) using feature augmentation by combining Model 1 and Model 2. There is an improvement in accuracy of more than 3% when compared to the constituent models. The fifth model was developed using the combination of Model 1 and Model 3, achieving an improvement in accuracy of more than 13%. This accuracy rate demonstrates that it is likely OF and EAR are the salient features that make lie recognition to be robust. Model 6 was developed using the combination of Model 2 and Model 3. Model 6 achieves an average recognition performance of 77.38%. In addition, Model six is better than the previous individual models and Model 4. We combined all the models as a single vector model in the ResNet-152-BLSTM, as explained in Section 3.3. This model achieves an accuracy of 92.14%. In addition, we employed a feature selection strategy to guide the BLSTM learning. With the keyframe selection algorithm, we developed a new model known as multi-STSTP, as illustrated in Table 8. The multi-STSTP model achieves an average recognition accuracy of 96.56%. We further compared the recognition accuracy of the proposed method with some state-of-the-art methods. 

### 5.1. Comparison between the ResNet-BLSTM and State-of-the-Art Methods 

For transparency, we selected some of the best methods with baseline features from real-life court trial videos. These methods were selected because they utilized the same data set as the proposed multi-STSTP method with ResNet-152-BLSTM. As shown in Table 9, this study analyzed the robustness of the two baseline features via hand gesture and face information. These two features are utilized by most of the existing works. The performance of these features shows that multi-feature fusion is highly needed to meet the required recognition results. For comparison, where a metric is not fully utilized by the method, it is represented with “NA”, which means that the metric is not assigned. However, the results of the proposed multi-STSTP method are in conformity with existing results in the literature, except for Krishnamurthy et al. [17] and Fisher-LSTM [1], whose feature performance is much better than ours. Krishnamurthy et al. achieved the best recognition performance of 64.97% and 80.79% for the individual features of the hand and face, respectively. When the two features were combined, the average recognition accuracy of the method was reduced drastically. Fisher-LSTM [1] was proposed to obtain temporal fine-grained information of hand and face poses, where each pose is treated as the energy density. The recognition performance of the Fisher-LSTM for individual features was superior to existing results. However, the average recognition results of the combined information decreased to 90.96%. The decrease in recognition performance was due to the Fisher-LSTM’s inability to capture most of the involuntary cues of people’s actions. The multi-STSTP method with the ResNet-152-BLSTM method was compared to the work in [4], and the results are presented in Table 10. This work employed LOSO cross-validation on real-life court trial videos, showing a recognition accuracy of 92.01% from the best classifier. However, the method reveals a precision using the AUC metric of 93.57%. We compared the performance results of the ResNet-152-BLSTM according to the recognition accuracy of the methods in [11,12,13,14,15,16,17,18,19,20,21,22,23,24,25]. Avola et al. [25] demonstrated that facial action units are good features for human RGB video recognition; however, these features are insufficient to discriminate between lie- and truth-based features. These findings prove that the combination of more than one feature can be harnessed to distinguish between the two classes of deception and truth. Therefore, the recognition accuracy and AUC of our proposed multi-STSTP method with ResNet-152-BLSTM outperformed most of the existing methods. The multi-STSTP vector model for the recognition of involuntary cognitive indices of a lying or truth-telling person is more reliable than the existing models.

### 5.2. Discussion 

**Figure 8:**
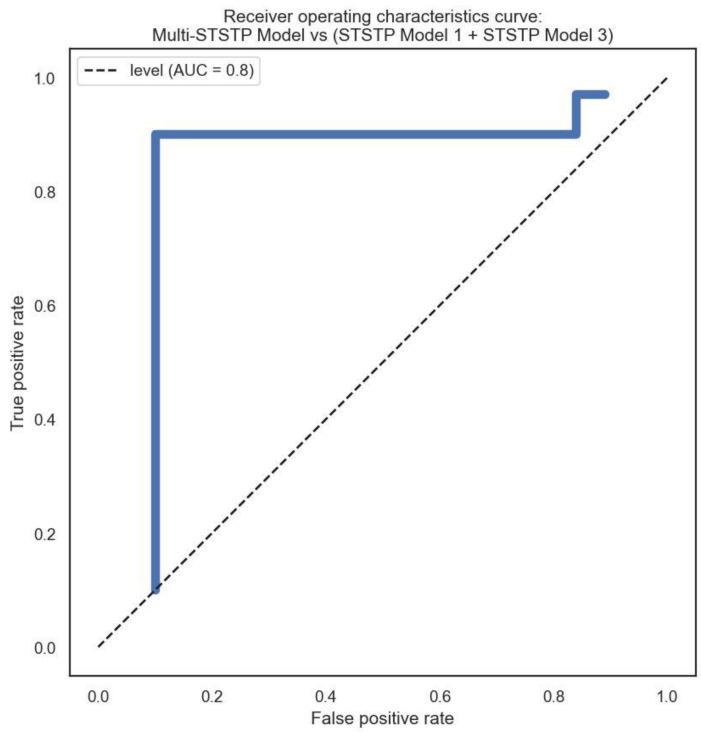
Sensitivity of the proposed multi-STSTP model.

**Figure 9:**
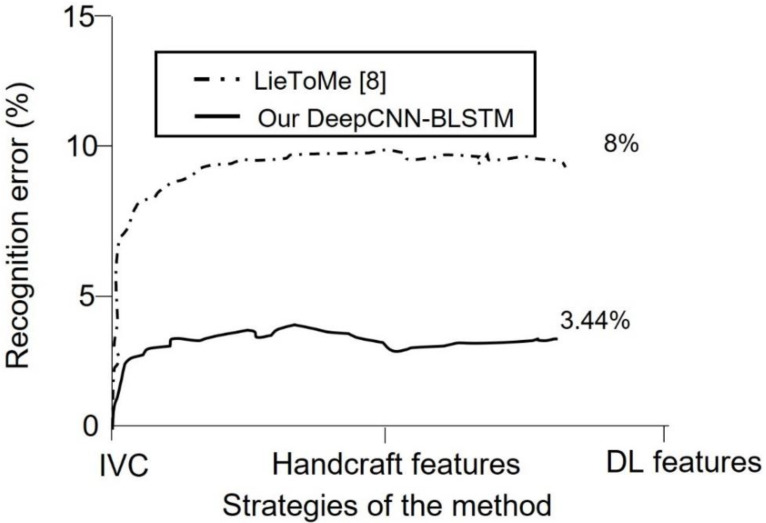
Recognition error of the proposed multi-STSTP with ResNet-152-BLSTM and an existing method.

We performed a keyframe selection to drop empty and noisy frames, as explained in Figure 1. The keyframe selection enhanced the feature extraction as well as the network learning. However, we conducted feature analysis using curve fitting tools to investigate the relationship and weight of each feature before training. The relationship of each feature from the curve fitting tool was obtained using a coefficient of determination metric. This approach of feature analysis provides a basis to learn the most significant features for model development. Furthermore, we conducted a qualitative analysis to learn the statistics of each demographic cue in lying or truth-telling actions. This analysis provides a basis for interviewers to concentrate more on some demographic data, while extracting important insights from the data. The analysis demonstrates that involuntary cognitive indices are present more in females than males, excluding hand actions. It also shows that eye blinking is the involuntary cognitive index that is performed more by all the demographic groups compared to other indices. It is observed that hand action is the index least performed by both two groups during lying. The involuntary cognitive indices lead to the acquisition of baseline features. We compared and analyzed baseline features that have appeared in most of the literature, as described in Table 9. The recognition performance of the proposed multi-STSTP model was performed in two phases. In the first phase, we designed a simple ResNet-152 network without any further optimization to achieve state-of-the-art performance. In the second phase, we designed a simple ResNet-152 based on the significant features of the proposed multi-STSTP model. The sensitivity of the best multi-STSTP model was obtained using the receiver operating characteristics curve (ROC), as illustrated in Figure 8. This study is interested in precisely recognizing a lie/deceptive class from court trial videos; thus, we performed a one-versus-one strategy that gives the sensitivity of the best features with a chance level of 0.8. The sensitivity of the network parameters on the proposed multi-STSTP features shows that the best model (multi-STSTP model) achieves a ROC of 97.58%, as shown in solid blue, while the second-best model (STSTP model 1 + STSTP model 2) achieves a ROC of 89.88%, as shown in the pre-step curves. The results obtained in Table 8 show that the multi-STSTP model achieves the best performance. We compared the recognition performance of the multi-STSTP model with one of the best existing recognition models, as shown in Figure 9. The recognition performance was compared based on the percentage of the recognition error versus the strategies employed by the recognition method, which include the basis of involuntary cues (IVC), the application of handcraft features, and the deep learning (DL) features. In addition, we compared the performance of the proposed multi-STSTP with the Fisher-LSTM in [1]. As shown in Table 9, the Fisher-LSTM outperforms our proposed method for the individual features of hand and face information. However, the average recognition of the combined hand and face information is not appreciable. The average recognition results show that our proposed multi-STSTP model is superior to existing lie recognition models, as well as the Fisher-LSTM. The major failure of the proposed approach is in two phases. The first phase of the failure is due to the EAR algorithm’s inability to compute the eye poses of highly noisy faces, such as the image of persons in scenes Lie036 and Truth041. The faces and eyes in these scenes are not upright. This problem could be addressed by using an adaptive approximation algorithm. The second phase of the failure is the inability of the PCA approximation to handle the issue associated with skin color in some video scenes, such as in Truth029. This failure could be addressed using the pixel-wise or fine-grain information approach. The proposed approach provides an effective model that could be built into a lie detection system to improve artificial intelligence detection of deception during police investigations, court trial processes, dispute resolution mechanisms, appropriate reward, and punishment mechanisms, etc.

## 6. Conclusions 

This work proposed a multi-STSTP method with hybrid Deep ResNet-152 and BLSTM to improve the performance of detecting lies in real-life court trial videos. The results show a significant correlation between hand, eye blinking, and body motion STSTP features, which were built inside the deep learning method for efficient recognition of involuntary cues from a lying or truth-telling person. This study initialized STSTP features using ResNet-152-BLSTM networks to handle complex long-time video frame estimation as potential enablers in conventional deep learning networks. Interestingly, the proposed method demonstrates outstanding performance in real-life court trial videos. Compared to some state-of-the-art methods, the proposed multi-STSTP method with ResNet-152-BLSTM achieves good recognition in the same data set because of the simple network design with the best parameter selection, which is automatically achieved from the feature space searching algorithm. In future work, we will design a single and automatic algorithm for all the methods for feature extraction so that the system can be extended to real-time applications by considering both the time complexity and the deployment issues.

## Figures and Tables

**Table 1:** Statistical confidence of eyebrow features using EAR.

	V1	V2	V3
V1	0.99999		
V2	0.99772	0.9992	
V3	0.99761	0.9974	0.99981

**Table 2:** Statistical confidence of eye features using EAR.

	Var1	Var2	Var3
Var1	0.99847		
Var2	0.99706	0.99465	
Var3	0.98863	0.9344	0.9965

**Table 3:** Confidence score between eye versus eyebrow features using EAR.

	Var1	Var2	Var3
V1	0.9858		
V2	0.95762	0.98768	
V3	0.9838	0.99291	0.98635

**Table 4:** Confidence test of the STSTP features.

	$\beta_{1}$	$\beta_{2}$	$\beta_{3}$
$\beta_{1}$	0.8334		
$\beta_{2}$	0.7641	0.8167	
$\beta_{3}$	0.7739	0.7992	0.8049

**Table 5:** Layers and parameters of the CNN-STSTP network.

No.	Layers Name	Activation
1	Image input	227 × 227 × 3
2	Convolution 1	55 × 55 × 3
3	ReLU 1	55 × 55 × 96
4	Cross Normalization 1	55 × 55 × 96
5	Max pooling 1	27 × 27 × 96
6	Convolution 2	27 × 27 × 256
7	ReLU 2	27 × 27 × 256
8	Cross Normalization 2	27 × 27 × 256
9	Max pooling 2	13 × 13 × 256
10	Convolution 3	13 × 13 × 384
11	ReLU 3	13 × 13 × 384
12	Convolution 4	13 × 13 × 384
13	ReLU 4	13 × 13 × 384
14	Convolution 5	13 × 13 × 256
15	ReLU 5	13 × 13 × 256
16	Max pooling 5	6 × 6 × 256
17	Fully Connected (Fc6)	1 × 1 × 4096
18	ReLU	1 × 1 × 4096
19	Dropout	1 × 1 × 4096
20	Fully Connected (Fc7)	1 × 1 × 4096
21	ReLU	1 × 1 × 4096
22	Dropout	1 × 1 × 4096
23	Fully Connected (Fc8)	1 × 1 × 1000
24	Softmax	1 × 1 × 1000
25	Classification Output	2 classes

**Table 6:** Layers and parameters of the ResNet-152-STSTP network.

	Conv7-64
	Maxpool
×3	Conv1-64S
	Conv3-64
	Conv1-256
×8	Conv1-128
	Conv3-128
	Conv1-512
×36	Conv1-256
	Conv3-256
	Conv1-1024
×3	Conv1-512
	Conv3-512
	Conv1-2048
	Conv1-2
	Global averaging pooling

**Table 7:** Suitable values for network training.

Network	Parameters	Values
ResNet-152	SGDM	0.9
	Batch size	128
	Max. iteration	500
	No. of epochs	250
	Gaussian with S.D	0.01
	Learning rate	0.01
	Weight decay	0.0005
	Dropout	0.7
	Params	>60 M
BLSTM	Input	1 dim.
	Hidden layer	100
	Output	Last
	Batch size	32
	FC	2
	Max epochs	64
	Dropout	0.2

**Table 8:** Model analysis.

Models	Feature Vector	Accuracy (%)
STSTP Model 1	OF	71.39
STSTP Model 2	Hand-PCA	59.87
STSTP Model 3	EAR	61.25
STSTP [Model 1 + Model 2]	OF + Hand-PCA	74.43
STSTP [Model 1 + Model 3]	OF + EAR	88.29
STSTP [Model 2 + Model 3]	Hand-PCA + EAR	77.38
**Multi-STSTP**	**OF + Hand-PCA + EAR**	**96.56**

**Table 9:** Comparison of different involuntary features from existing methods.

Methods	Hand Gesture		Facial Information	
	AUC (%)	Accuracy (%)	AUC (%)	Accuracy (%)
RF [12]	NA	62.8	NA	76.03
DT [12]	NA	71.9	NA	59.5
L-SVM [15]	NA	NA	66.33	NA
MLP [17]	66.71	64.97	94.16	80.79
Fisher-LSTM [1]	91.14	90.96	NA	NA
ResNet-152-BLSTM	68.09	59.87	73.11	61.25

**Table 10:** Comparison between multi-STSTP ResNet-152-BLSTM with some state-of-the-art methods.

Methods	AUC (%)	Accuracy (%)
RF [12]	NA	75.2
DT [12]	NA	50.41
L-SVM [15]	90.65	NA
LR [15]	92.21	NA
Hierarchical-BSSD [16]	67.1	NA
LSTM [16]	66.5	NA
RBF-SVM [25]	NA	76.84
Fisher-LSTM [1]	91.14	90.96
Stacked MLP [4]	93.57	92.01
NN [5]	94	84.18
**ResNet-152-BLSTM**	**97.58**	**96.56**

## Data Availability

The data are obtained upon request from the work [1].

## Abbreviations

The following abbreviations are used in this manuscript:

STSTP	Spatial–temporal state transition patterns
BLSTM	Bidirectional long short-term memory
CV	Computer vision
DL	Deep learning
ML	Machine learning
RGB	Red, green, and blue
CNN	Convolutional neural network
SVM	Support vector machine
LUT	Look-up table
EAR	Eye aspect ratio
ResNet	Residual network
PCA	Principal component analysis
OF	Optical flow
FC	Fully connected layer.
POI	Point of interest
CHT	Circular Hough transform
SGDM	Stochastic gradient decent with momentum
ReLU	Rectified linear unit.
IVC	Involuntary cognitive cues

## References

[1] Avola D., Cinque L., Maria D., Alessio F., Foresti G. (2020). LieToMe: Preliminary study on hand gestures for deception detection via Fisher-LSTM. Pattern Recognit. Lett..

[2] Al-jarrah O., Halawan A. (2001). Recognition of gestures in Arabic sign language using neuro-fuzzy systems. Artif. Intell..

[3] Abdullahi S.B., Khunpanuk C., Bature Z.A., Chroma H., Pakkaranang N., Abubakar A.B., Ibrahm A.H. (2022). Biometric Information Recognition Using Artificial Intelligence Algorithms: A Performance Comparison. IEEE Access.

[4] Avola D., Cascio M., Cinque L., Fagioli A., Foresti G. (2021). LieToMe: An Ensemble Approach for Deception Detection from Facial Cues. Int. J. Neural Syst..

[5] Sen U., Perez V., Yanikoglu B., Abouelenien M., Burzo M., Mihalcea R. (2022). Multimodal deception detection using real-life trial data. IEEE Trans. Affect. Comput..

[6] Ding M., Zhao A., Lu Z., Xiang T., Wen J. (2019). Face-focused cross-stream network for deception detection in videos. Proceedings of the IEEE/CVF Conference on Computer Vision and Pattern Recognition.

[7] Abdullahi S.B., Chamnongthai K. (2022). American sign language words recognition using spatio-temporal prosodic and angle features: A sequential learning approach. IEEE Access.

[8] Bhaskaran N., Nwogu I., Frank M.G., Govindaraju V. (2011). Lie to me: Deceit detection via online behavioral learning. Proceedings of the 2011 IEEE International Conference on Automatic Face & Gesture Recognition (FG).

[9] Jeffry G., Jenkins J.L., Burgoon J.K., Judee K., Nunamaker J.F. (2015). Deception is in the eye of the communicator: Investigating pupil diameter variations in automated deception detection interviews. Proceedings of the 2015 IEEE International Conference on Intelligence and Security Informatics (ISI).

[10] Thakar M.K., Kaur P., Sharma T. (2022). Validation studies on gender determination from fingerprints with special emphasis on ridge characteristics. Egypt. J. Forensic Sci..

[11] Meservy T.O., Jensen M.L., Kruse J., Burgoon J.K., Nunamaker J.F., Twitchell D.P., Tsechpenakis G., Metaxas D.N. (2005). Deception detection through automatic, unobtrusive analysis of nonverbal behavior. IEEE Intell. Syst..

[12] Pérez-Rosas V., Abouelenien M., Mihalcea R., Burzo M. (2015). Deception detection using real-life trial data. Proceedings of the 2015 ACM on International Conference on Multimodal Interaction.

[13] Abouelenien M., Pérez-Rosas V., Mihalcea R., Burzo M. (2017). Detecting deceptive behavior via integration of discriminative features from multiple modalities. IEEE Trans. Inf. Forensics Secur..

[14] Karimi H., Tang J., Li Y. (2018). Toward end-to-end deception detection in videos. Proceedings of the 2018 IEEE International Conference on Big Data (Big Data).

[15] Wu Z., Singh B., Davis L., Subrahmanian V. (2018). Deception detection in videos. Proceedings of the AAAI conference on artificial intelligence.

[16] Rill-García R., Jair E.H., Villasenor-Pineda L., Reyes-Meza V. (2019). High-level features for multimodal deception detection in videos. Proceedings of the IEEE/CVF Conference on Computer Vision and Pattern Recognition Workshops.

[17] Krishnamurthy G., Majumder N., Poria S., Cambria E. (2018). A deep learning approach for multimodal deception detection. arXiv.

[18] Abdullahi S.B., Ibrahim A.H., Abubakar A.B., Kambheera A. (2022). Optimizing Hammerstein-Wiener Model for Forecasting Confirmed Cases of COVID-19. IAENG Int. J. Appl. Math..

[19] Abdullahi S.B., Muangchoo K. (2020). Semantic parsing for automatic retail food image recognition. Int. J. Adv. Trends Comput. Sci. Eng..

[20] Lu S., Tsechpenakis G., Metaxas D.N., Jensen M.L., Kruse J. (2005). Blob analysis of the head and hands: A method for deception detection. Proceedings of the 38th Annual Hawaii International Conference on System Sciences.

[21] Abdullahi S.B., Chamnongthai K. (2022). American sign language words recognition of skeletal videos using processed video driven multi-stacked deep LSTM. Sensors.

[22] Abdullahi S.B., Chamnongthai K. (2021). Hand pose aware multimodal isolated sign language recognition. Multimed. Tools Appl..

[23] Reddy B., Kim Y., Yun S., Seo C., Jang J. (2017). Real-Time Eye Blink Detection using Facial Landmarks. Proceedings of the Conference on Computer Vision and Pattern Recognition.

[24] He K., Zhang X., Ren S., Sun J. (2016). Deep residual learning for image recognition. Proceedings of the Conference on Computer Vision and Pattern Recognition.

[25] Avola D., Cinque L., Foresti G.L., Pannone D. (2019). Automatic deception detection in rgb videos using facial action units. Proceedings of the 13th International Conference on Distributed Smart Cameras.

